# β-Sitosterol-D-Glucopyranoside Mimics Estrogenic Properties and Stimulates Glucose Utilization in Skeletal Muscle Cells

**DOI:** 10.3390/molecules26113129

**Published:** 2021-05-24

**Authors:** Jyotsana Pandey, Kapil Dev, Sourav Chattopadhyay, Sleman Kadan, Tanuj Sharma, Rakesh Maurya, Sabyasachi Sanyal, Mohammad Imran Siddiqi, Hilal Zaid, Akhilesh Kumar Tamrakar

**Affiliations:** 1 Biochemistry Division, CSIR-Central Drug Research Institute, Lucknow 226031, India; jyotsna900@gmail.com (J.P.); souravthezidane@rediffmail.com (S.C.); sanyal@cdri.res.in (S.S.); 2 Academy of Scientific and Innovative Research (AcSIR), Ghaziabad 201002, India; 3 Medicinal and Process Chemistry Division, CSIR-Central Drug Research Institute, Lucknow 226031, India; kapildeopatel@gmail.com (K.D.); mauryarakesh@rediffmail.com (R.M.); 4 Qasemi Research Center, Al-Qasemi Academic College, P.O. Box 124, Baqa El-Gharbia 30100, Israel; slemanka@gmail.com; 5 Molecular and Structural Biology Division, CSIR-Central Drug Research Institute, Lucknow 226031, India; tanush84@gmail.com (T.S.); mi_siddiqi@cdri.res.in (M.I.S.); 6 Faculty of Sciences and Faculty of Medicine, Arab American University, P.O. Box 240, Jenin 009704, Palestine

**Keywords:** *Cupressus sempervirens*, phytoestrogen, glucose uptake, insulin resistance, skeletal muscle

## Abstract

Estrogenic molecules have been reported to regulate glucose homeostasis and may be beneficial for diabetes management. Here, we investigated the estrogenic effect of *β-*sitosterol-3-O-D-glucopyranoside (BSD), isolated from the fruits of *Cupressus sempervirens* and monitored its ability to regulate glucose utilization in skeletal muscle cells. BSD stimulated ERE-mediated luciferase activity in both ERα and ERβ-ERE luc expression system with greater response through ERβ in HEK-293T cells, and induced the expression of estrogen-regulated genes in estrogen responsive MCF-7 cells. In silico docking and molecular interaction studies revealed the affinity and interaction of BSD with ERβ through hydrophobic interaction and hydrogen bond pairing. Furthermore, prolonged exposure of L6-GLUT4*myc* myotubes to BSD raised the glucose uptake under basal conditions without affecting the insulin-stimulated glucose uptake, the effect associated with enhanced translocation of GLUT4 to the cell periphery. The BSD-mediated biological response to increase GLUT4 translocation was obliterated by PI-3-K inhibitor wortmannin, and BSD significantly increased the phosphorylation of AKT (Ser-473). Moreover, BSD-induced GLUT4 translocation was prevented in the presence of fulvestrant. Our findings reveal the estrogenic activity of BSD to stimulate glucose utilization in skeletal muscle cells via PI-3K/AKT-dependent mechanism.

## 1. Introduction

Type 2 diabetes mellitus is a chronic metabolic disorder associated with impaired glucose and lipid metabolism. The pandemic development of metabolic disorders warrants a better understanding of the factors regulating lipid and glucose metabolism for the better management of the associated complications. Over the past few years, emerging evidence signifies the role of estrogens as an important regulator of glucose and lipid metabolism [1]. Estrogens are known to modulate fat distribution and metabolism, and insulin sensitivity. In support of this notion, mice deficient in estrogen receptor gene display features of metabolic syndrome such as increased adiposity, glucose intolerance, and insulin resistance [2], demonstrating the implication of estrogens in nutrient metabolism.

Estrogens exert their biological effects through binding to their receptors which exist in two isoforms, ERα and ERβ. Estrogen receptors (ERs) are distributed throughout the body, displaying tissue and cell-type specific expression [3,4]. In insulin sensitive metabolic tissues, both isoforms of ER are expressed [5,6] in a distinct grade. In skeletal muscle, ERβ predominates, whereas, in adipose tissue, ERα is the predominating isoform [7]. Typically, estrogens exert their biological action via nuclear ERs, which directly bind to specific estrogen responsive elements (EREs) in the promoter region of target genes [3]. Additionally, estrogen has been established to exert extra nuclear effects via classical ERs presented at the plasma membrane [8], alternative spliced variant of ERα [9], or via G protein-coupled estrogen receptor (GPER1) [10]. These extra nuclear effects involve the activation of certain signaling cascade, including the PI-3-K/AKT pathway [11,12]. Since PI-3-K/AKT participates in the insulin signaling cascade, estrogens might play an important role in regulating nutrient metabolism in insulin sensitive tissues. Estrogens have been reported to modulate insulin-stimulated glucose uptake in adipocytes [13,14]. Moreover, ER deficient mice displayed altered GLUT4 expression and glycemic homeostasis [5] and selective ERβ activation stimulates skeletal muscle growth and regeneration [15]. Overall, it seems that estrogens via extra nuclear mechanisms might regulate glucose homeostasis in insulin-sensitive metabolic tissues.

However, being a steroid hormone with a prominent effect on female reproductive physiology, therapeutic use of estrogen for the management of glucose homeostasis has limitations. To exploit the beneficial potential of estrogens without having side effects, plant-based estrogens like molecules (Phytoestrogens) may be potential intervention for the management of glucose homeostasis. Phytoestrogens are non-steroidal compounds of natural origin that behave as estrogen mimics and can act as ligands to estrogen receptors [16]. Numerous studies in human and experimental animals suggest the beneficial role of phytoestrogens in improving glucose homeostasis and insulin sensitivity. Isoflavone consumption in humans is associated with reduction in BMI and serum insulin levels [17] and dietary phytoestrogens improve insulin sensitivity in ovariectomizedcynomolgus monkeys [18]. Similarly, consumption of phytoestrogen-rich diet has been shown to increase energy expenditure and decrease adiposity in mice [19]. Overall, phytoestrogens, through their estrogen-like effects, may regulate glucose metabolism and insulin sensitivity and might contribute to the management of peripheral insulin resistance and its consequences.

Insulin resistance plays a prominent role in pathophysiology of type 2 diabetes and is characterized by diminished activity of insulin to regulate nutrient metabolism in target tissues, including liver, skeletal muscle, and adipose. The prime site for the postprandial glucose utilization is the skeletal muscle where the uptake of glucose is the rate limiting step for its utilization [20]. In skeletal muscle cells, acceleration of the rate of translocation and redistribution of the insulin sensitive glucose transporter 4 (GLUT4) to the cell periphery is a typical feature for increased glucose uptake [21], and the process gets compromised under insulin resistance, leading to a decrease in glucose uptake potential. Since insulin resistance is the early abnormality in the pathogenesis of type 2 diabetes, there has been ample interest for characterizing insulin-sensitizing agents for the disease management [22,23,24]. With our interest to explore phytoestrogens for the management of glucose homeostasis, β-sitosterol-3-O-D-glucopyranoside (BSD) was isolated from *Cupressus sempervirens* fruits and characterized with the help of NMR and mass spectrometric analysis. It contains an aglycone moiety (β-sitosterol) attached with glycone (D-glucose) at its 3rd position and a double bond at the 5th position. β-sitosterol was previously reported for their plasma cholesterol lowering effect as well as anti-diabetic and antioxidant potentials in a streptozotocin-induced hyperglycemic model [25,26], whereas β-sitosterol-3-O-D-glucopyranoside (BSD) was reported for insulin secretary effects [27]. Given the ability of phytoestrogens to modulate glucose metabolism, we hypothesized to characterize phytoestrogenic activity of BSD and to explore its ability to regulate glucose utilization in skeletal muscle cells.

## 2. Materials and Methods

### 2.1. Materials

Cell culture medium (DMEM), cytochalasin B, protease inhibitor cocktail, polyclonal anti-*myc* antibody, Estradiol, 4,4′,4”-(4-propyl-[1H]-pyrazole-1,3,5-triyl) trisphenol (PPT), 2,3-bis(4-hydroxy-phenyl)-propionitrile (DPN), fulvestrant, and all other chemicals, unless noted otherwise, were from Sigma Chemical (St. Louis, MO, USA). Fetal bovine serum (FBS), antibiotic/antimycotic solution, trypsin, and charcoal stripped fetal bovine serum (CSFBS) were from Gibco (USA). 2-Deoxy-D-[^3^H]-glucose (2-DG) was from PerkinElmer (Boston, MA, USA). Antibody to phospho-AKT (Ser-473), AKT, and HRP-conjugated secondary antibody were from Cell Signaling Technology (Danvers, MA, USA).

### 2.2. Plant Material

The fruits of *C. sempervirens* were collected from Garhwal, Uttrakhand, India in the month of March 2012 and identified by the Botanist in the Botany Division of CSIR-Central Drug Research Institute, Lucknow. The voucher specimen (No. 24421) is preserved in the herbarium of the Institute. The fresh fruits were dried under shade and powdered for further extraction.

### 2.3. Extraction, Isolation, and Characterization of β-Sitosterol-D-Glucopyranoside(BSD)

**Figure 1:**
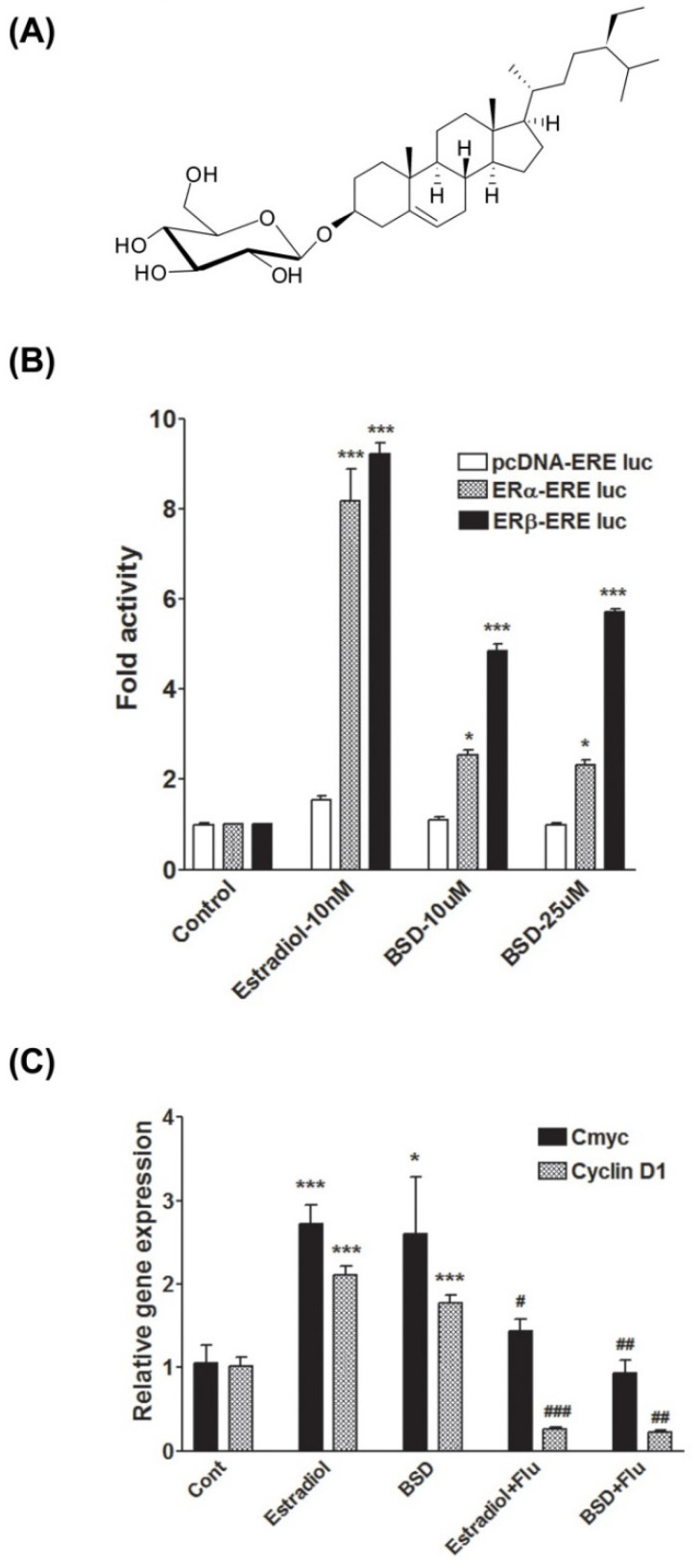
(**A**) Chemical structure of β-sitosterol-D-glucopyranoside (BSD) isolated from the fruits of *C. sempervirens*; (**B**) estrogenic activity of BSD in ERα/β-ERE luc expression system in HEK-293T cells. Cells were transiently transfected with Estrogen response element-luc reporter (ERE-Luc) and ERα/β containing plasmids and incubated with indicated concentrations of BSD or Estradiol. Cells were lysed, and relative luciferase activity was determined. Results are expressed as fold over control. Results shown are mean ± SE of three independent experiments. * *p* < 0.05, *** *p* < 0.001 relative to respective control condition; (**C**) effect of BSD (10 µM)on expression of ER responsive genes in MCF-7 cells. Cells were treated as indicated for 24 h and mRNA expression level of C-myc and cyclin D1 was determined by quantitative real-time PCR. Results are expressed as fold change over control, *n* = 4. * *p* < 0.05, *** *p* < 0.001 relative to control, # *p* < 0.05, ## *p* < 0.01,### *p* < 0.001 relative to respective without a Fulvestrant treated condition.

The dried and powdered fruits (4.00 kg) of *C. sempervirens* were percolated three times successively with 95% ethanol at room temperature. The combined extract was filtered and concentrated under reduced pressure at 40–45 °C afforded dark brown residue (400 g). Ethanol extract (300 g) was triturated with hexane (4 × 600 mL), dried under vacuum and afforded hexane fraction (50 g). The hexane insoluble portion was dissolved in distilled water (500 mL), which was successively extracted with chloroform (4 × 500 mL) and *n*-butanol (4 × 500 mL) and yielded fractions of chloroform (60 g), *n*-butanol (90 g) and water (100 g). *n*-butanol fraction (80 g) was subject to column chromatography over silica gel (100–200 mesh) and eluted with gradient systems of increasing polarity chloroform: methanol (0–100%) and finally with methanol. Twenty fractions were collected and combined on the basis of their TLC profiles to afford 10 fractions, F1–F10. Fraction F3 was further purified on silica gel (230–400 mesh) by using chloroform: methanol (85:15) as eluent, yielded compound **1**(180 mg, 0.06%). This compound was further purified by crystallization in a chloroform–methanol mixture with the HPLC purity 98.7%. The compound was characterized as *β-*sitosterol-3-O-D-glucopyranoside (BSD, Figure 1A) with the help of 1D, 2D NMR spectra and mass spectrometry analysis [28]. The compound was isolated as an off white solid; FTIR (KBr; *υ_max_,* cm^−1^) 3535, 3420, 2950, 2410, 1650, 1460, 1384, 1378, 920, 762, 660, 620. The ESIMS spectrum showed an [M+H]^+^ ion peak at *m*/*z* 578 corresponding to molecular formula of C_35_H_61_O_6_.^1^H NMR (400 MHz, Pyridine-*d*_5_) δ_ppm_; 0.66 (*s*, 3H, H-18), 0.87 (*t*, *J* = 7.6 Hz, 3H, H-29), 0.84–0.86 (*m*, 3H), 0.90 (*d*, *J* = 7.3 Hz, 3H, H-21), 0.93 (*s*, 3H, H-19), 0.98 (*d*, *J* = 6.4 Hz, 6H), 1.06–1.13 (*m*, 4H), 1.22–1.30 (*m*, 5H), 1.32–1.46 (*m*, 6H), 1.50–1.57 (*m*, 2H), 1.64–1.76 (*m*, 3H), 1.82–1.92 (*m*, 2H), 1.96–1.99 (*m*, 1H, H-7), 2.13 (*d*, *J* = 11.8 Hz, 1H, H-4) 2.44–2.50 (*m*, 1H, H-7), 2.72 (*dd*, *J* = 13.3, 2.5 Hz, 1H, H-4), 3.89–4.01 (*m*, 2H, H-6′, H-3), 4.05 (*t*, *J* = 8.07 Hz, 1H, H-6′), 4.23-4.32 (*m*, 2H, H-4′, H-5′), 4.39 (*dd*, *J* = 11.6, 4.7 Hz, 1H, H-3′), 4.55 (*dd*, *J* = 11.7, 1.8 Hz, 1H, H-2′), 5.04 (*d*, *J* = 7.8 Hz, 1H, H-1′), 5.33–5.36 (*m*, 1H, H-6);^13^C NMR (100 MHz, Pyridine-*d_5_*) δ_ppm_; 11.7 (C-18), 11.9 (C-29), 18.8 (C-21), 19.0 (C-26), 19.2 (C-19), 19.7 (C-27), 21.0 (C-11), 23.2 (C-28), 24.3 (C-15), 26.2 (C-23), 28.3 (C-16), 29.2 (C-25), 30.0 (C-2), 31.8 (C-7), 31.9 (C-8), 34.0 (C-22), 36.1 (C-20), 36.7 (C-10), 37.2 (C-1), 39.1 (C-4), 39.7 (C-12), 42.2 (C-13), 45.8 (C-24), 50.1 (C-9), 56.0 (C-17), 56.6 (C-14), 62.6 (C-6′), 71.4 (C-4′), 75.10 (C-2′), 77.9 (C-3), 78.2 (C-3′), 78.3 (C-5′), 102.3 (C-1′), 121.7 (C-6), 140.7 (C-5).

### 2.4. Cell Culture

Human MCF-7 and HEK-293T cells were cultured in DMEM high glucose, supplemented with 10% fetal bovine serum (FBS) and 1% antibiotic/antimycotic solution containing 10,000 U/mL penicillin G, 10 mg/mL streptomycin, 25 μg/mL amphotericin B, in a humidified atmosphere of air and 5% CO_2_ at 37 °C. L6 rat skeletal muscle cells stably expressing rat GLUT4 with a *myc* epitope inserted in the first exofacial loop (L6-GLUT4*myc*) were a kind gift of Amira Klip, Program in Cell Biology, The Hospital for Sick Children, Toronto, Canada. Cells were maintained in DMEM supplemented with 10% FBS and 1% antibiotic/antimycotic solution in a humidified atmosphere of air and 5% CO_2_ at 37 °C [29]. Cells were allowed to differentiate into myotubes by replacing the medium supplemented with 2% FBS. Experiments were performed in differentiated myotubes 5–6 days after seeding.

### 2.5. Transfections and Luciferase Assays

The BSD was evaluated for its ability to activate estrogen receptor α and β in cell-based luciferase assays using HEK-293T cells. Estrogen responsive elements (ERE)-Luc, pcDNAERα, and pcDNAERβ were kind gifts from Eckardt Treuter, Department of Biosciences and Nutrition, Karolinska Institutet, Stockholm, Sweden. For transient-transfection, HEK-293T cells were seeded onto 48-well plates in phenol red free DMEM supplemented with 10% charcoal stripped FBS. After 24 h of seeding, cells were co-transfected with 0.15 µg Estrogen response element-luc reporter (ERE-Luc), 0.15 µg ERα/β plasmids using Lipofectamine LTX with plus reagent (Life Technologies, Carlsbad, CA, USA) according to manufacturer’s instructions. Total DNA in each transfection were kept constant to 350 ng/well using pcDNA3 empty vector. In all transfections, pEGFPC1 was used as internal controls. Twenty-four hours after transfection, cells were treated with Estradiol (10 nM) as positive control or BSD at 10 µM and 25µM concentrations. After 24 h of treatment, cells were lysed and relative luciferase activity was determined using StayBrite™ D-Luciferin (Biovision Inc., Milpitas, CA, USA) substrate in a GloMax-96 Microplate luminometer (Promega) and GFP fluorescence was quantified using a multiplate fluorimeter (POLARstar Galaxy; BMG Labtech, Cary, NC, USA). Luciferase values were then normalized with GFP values and were plotted as fold activities over untreated controls.

### 2.6. Glucose Uptake Assay

The rate of glucose uptake was determined by 2-Deoxy-D-[^3^H]-glucose (2-DG) transport in L6-GLUT4*myc*myotubes. Differentiated myotubes were treated with BSD as indicated and glucose uptake measurement was performed by incubating cells for 10 min in HEPES-buffered saline containing 10 µM 2-DG (0.5 µCi/mL 2-[^3^H] DG) at room temperature, followed by cell lysis and measurement of radioactivity incorporated by scintillation counting [30]. Nonspecific uptake was determined in the presence of cytochalasin B (25 µM) during the assay.

### 2.7. GLUT4 Translocation Assay

The surface GLUT4 level in non-permeabilized L6-GLUT4*myc* myotubes was measured by an antibody-coupled colorimetric assay [29]. After indicated treatments with BSD, myotubes were fixed in 3% paraformaldehyde and quenched in 100 mM glycine. After the blocking in 5% FBS, cells were incubated with the anti-*myc* primary antibody solution for 1 h followed by washing with PBS to remove excess labeling. The bound antibody was probed by HRP-conjugated secondary antibodies followed by the detection of bound HRP by an O-phenylenediaminedihydrochloride reagent.

### 2.8. Animal

Female SD rats, aged 7–8 weeks, available at the National Laboratory Animal Center of the CSIR-Central Drug Research Institute, Lucknow were used for the study. The work with these animals was cleared by Institutional Animal Ethics Committee (IAEC) of the Institute and was conducted in accordance with the guidelines of the Committee for the purpose of Control and Supervision of Experiments on Animals (CPCSEA) formed by the Government of India. Rats were housed under standard conditions of temperature 23 ± 2 °C with relative humidity (50–60%), light 300 Lx at floor level along with light and dark cycles of 12 h. Animals were provided with a standard diet and drinking water ad libitum. After acclimatization, animals were killed by cervical dislocation and skeletal muscle and ovary tissues were excised out and used for RNA isolation.

### 2.9. Western Blot Analysis

L6-GLUT4*myc* myotubes were treated with BSD and following cell lysis in RIPA buffer, protein samples were prepared and resolved by SDS-PAGE and transferred to polyvinylidenedifluoride (PVDF) membrane using electrophoresis and blotting system (Bio-Rad). The membranes were incubated with antibodies to phospho-AKT (Ser-473)/total AKT, followed by incubation with HRP-conjugated secondary antibodies. Immunoreactive bands were visualized by Enhanced Chemiluminescence system (Thermo Scientific) and further calculated using National Institute of Health (NIH) ImageJ software.

### 2.10. Gene Expression Analysis

Cells were treated as indicated, and total RNA was extracted using guanidine isothiocyanate-phenol-cholorofrom (TRIZOL). RNA concentration was assessed spectrophotometrically using the Nano Drop ND-1000 Spectrophotometer (Thermo Scientific). The ratio of absorbance at 260/230 and at 260/280 was used as indicators for RNA purity. RNA was reverse transcribed to generate cDNA using a Verso cDNA Synthesis Kit (Thermo Scientific). For semi-quantitative analysis, cDNA was amplified using a Taq PCR core kit using gene specific primers. Quantitative real-time PCR was carried out using a CYBR green master mix (DyNAmo Flash SYBR Green qPCR Kit) following manufacturer’s instruction on the CFX96TM real-time system (Biorad, Hercules, CA, USA). All quantifications were performed with 18S RNA as internal control and the relative amount of mRNA was presented in the form of fold change over control. The primers used in the study were designed and validated using Universal Probe Library. The concentration of the primer in the reaction was 1μM and the length of the product ranges from 70–80 bp. The expression data were calculated on the basis of ct value obtained from quantitative real-time PCR. The primer sequences used are shown in Table 1.

### 2.11. Molecular Docking Study

Molecular interactions of BSD with estrogen receptor protein were studied by developing a receptor model having PDBid 2J7X for computational studies. Binding pocket was generated using a SiteMap module of the Schrodinger package [31]. Furthermore, the grid around protein was generated using a Grid generating module of the Schrodinger software package [32]. Van der Waals radius used for scaling was 1.0 Å and default settings were used for other parameters. Both protein and ligands were prepared using the Protein and Ligand preparation module of the Schrodinger software package [33]. Prepared ligands were then docked with Estradiol using a Glide-7.1 module [32,34]. Docking was performed using the extra precision mode and default settings were used for docking. Images were generated using the Ligand–Receptor interaction module of the Schrodinger software package. The interactions which were being analyzed in this module were Hydrogen Bonding, Pi–Pi interaction, Pi–Cation interaction; Salt Bridges at cut off radius of 2.50 Å. Interaction images were generated using a ‘Ligand–interaction’ module of Schrodinger software package and UCSF Chimera [35].

### 2.12. Statistical Analysis

Values are given as mean ± SEM. Analysis of statistical significance of differences in measurements between samples was done by one-way ANOVA with Dunnets post hoc test (GraphPad Prism version 3). *p* < 0.05 was considered statistically significant.

## 3. Results

### 3.1. Phytoestrogenic Activity of BSD

To identify the estrogenic potential, BSD was assayed on estrogen receptor α and estrogen receptor β dependent activity in HEK-293T cells transiently transfected with plasmids containing either ERα or ERβ and an estrogen-responsive element (ERE)-fused luciferase reporter plasmid (ERα/β-ERE luc). Estradiol (10 nM) was used as positive control, which showed a profound increase in luciferase reporter gene activity in both ERα-ERE luc and an ERβ-ERE luc expression system. In the ERα-ERE luc expression system, BSD stimulated ERE-mediated luciferase activity by around 2.5-fold (*p* < 0.05) at both 10 and 25 μM concentrations. In the ERβ-ERE luc expression system, BSD dose-dependently induced ERE-mediated luciferase activity by 4.8 and 5.7-fold increase at 10 and 25 μM concentrations, respectively (Figure 1B). Results suggested that BSD possesses estrogenic activity and exerts its estrogenic action preferentially through ERβ.

To further characterize the estrogenic response of BSD, we investigated the effect of BSD on the expression of estrogen-regulated genes: *c-Myc* and *Cyclin D1*, which are known as estrogen inducible genes, in MCF-7 human breast cancer cells. As depicted in Figure 1C, Estradiol (10 nM) treatment upregulated the mRNA expression of both *c-Myc* and *Cyclin D1*. Similar to Estradiol, treatment with BSD significantly increased the mRNA expression of *c-Myc* and *cyclin D1* in MCF-7 cells (Figure 1C). The presence of fulvestrant (500 nM), a pharmacological inhibitor of estrogen receptor, inhibited the Estradiol-or BSD-induced mRNA expression of *c-myc* and *cyclin D1*. Together, these data suggest the estrogenic effect of BSD. Therefore, we performed molecular docking studies in silico to characterize the interaction of BSD with the ER LBD.

### 3.2. Molecular Interaction Study

Furthermore, using the estrogen receptor-β protein structure (PDB-id-2J7X) of *Rattus norvegicus*, molecular docking was performed using GLIDE-7.1. Docking studies illustrated that BSD has high binding affinity towards the estrogen receptor-β, compared to the Estradiol. The glide score for BSD and Estradiol was observed as −9.42 and −8.95 (Table 2), respectively, validating the molecular interaction of BSD with an estrogen receptor.

**Figure 2:**
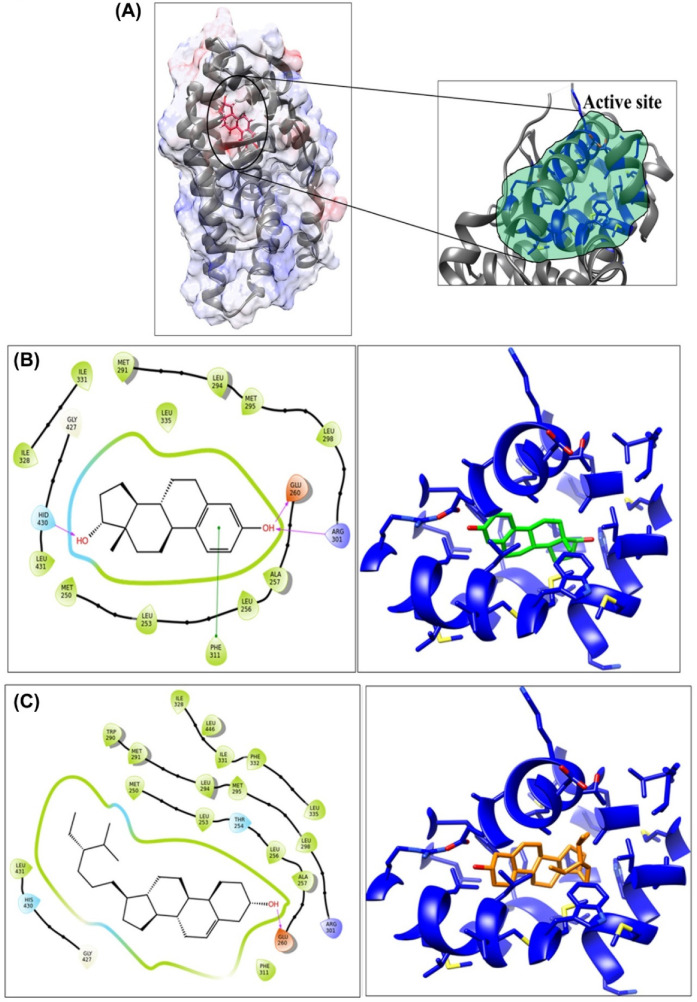
The Estradiol bound receptor structure of estrogen receptor β (**A**). The 2D plot diagram along the 3D conformation of the BSD (**B**) and Estradiol (**C**) molecule, along with the bound receptor structure.

Molecular interaction studies revealed that the residues which were involved in interaction with BSD were Met-250, Leu-253, Thr-254, Leu-256, Ala-257, Glu-260, Trp-290, Met-291, Leu-294, Met-295, Leu-298, Arg-301, Phe-311, Ile-328, Ile-331, Phe-332, Leu-335, Gly-427, His-430, Leu-431, and Leu-446 (Figure 2B). Of these, most of the residues were hydrophobic and were involved in hydrophobic interaction with the non-polar ethyl-methyl-hexyl chain of the molecule. In addition, a prominent hydrogen bond pairing with the carboxylic side chain of Glu-260 was observed with the hydroxyl group of phenanthrenol fragment. For Estradiol, the residues involved in interaction were Met-250, Leu-253, Leu-256, Ala-257, Glu-260, Met-291, Leu-294, Met-295, Leu-298, Arg-301, Phe-311, Ile-328, Ile-331, Leu-335, Gly-427, His-430, and Leu-431 (Figure 2C).

Hydrogen bond interactions were observed with side chain of Glu-260 and Arg-301, involving hydroxyl group of phenanthrenol fragment on one side and with His-430 on other side. In addition, pi–pi interactions were observed with aromatic residue Phe-311. In summary, hydrophobic interactions of the BSD molecule plays a crucial role in increasing the binding affinity with the receptor.

### 3.3. Skeletal Muscle Expresses Both Estrogen Receptor α and β

**Figure 3:**
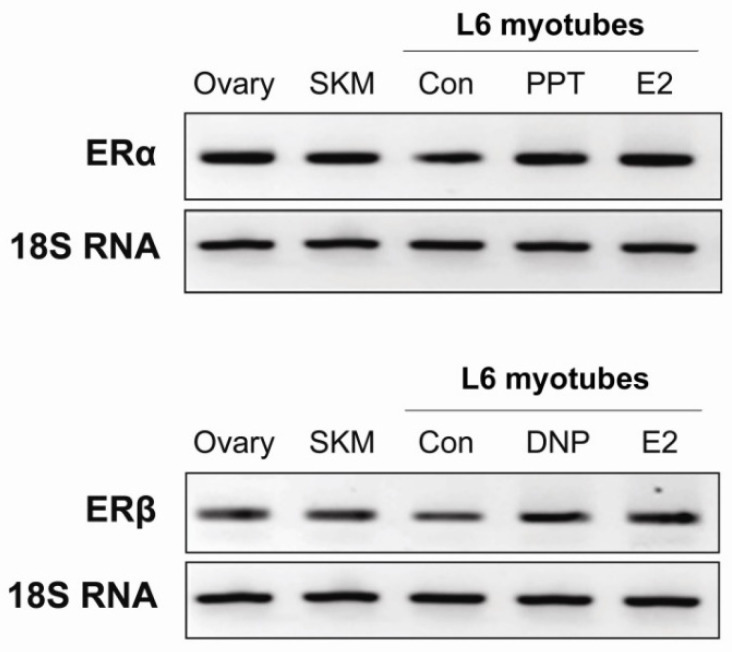
Expression of estrogen receptor α and β in rat skeletal muscle tissue and L6 skeletal muscle cells. Total RNA was extracted from the ovary and skeletal muscle (SKM) tissue of the rat, and L6 myotubes treated with 4,4′,4”-(4-propyl-[1H]-pyrazole-1,3,5-triyl) trisphenol (PPT), 2,3-bis(4-hydroxy-phenyl)-propionitrile (DPN) orEstradiol (E2). RNA was reverse transcribed to generate cDNA followed by semi-quantitative analysis using gene specific primers.

Skeletal muscle, being the major insulin-targeted tissue, is responsible for maintenance of whole body glucose homeostasis and contributing to the almost 80% of the glucose utilization [36]. Thus, we aimed to investigate the effect of BSD on glucose utilization in skeletal muscle. We used L6 rat skeletal muscle cells as model system and first checked for the expression of estrogen receptors in skeletal muscle. The template minus sample was included as a negative control in the experiment which did not show any expression of ERα or ERβ genes. The ovary tissue from the rat was taken as a positive control, which showed significant expression of both ERα and ERβ isoforms (Figure 3). We found that rat skeletal muscle and our L6 skeletal muscle cell lines at the myotubes stage express both α and β isoforms of estrogen receptor. Importantly, treatment with Estradiol (10 nM) or specific agonists of ERα and ERβ, namely PPT (100 nM) and DNP (100 nM), respectively upregulated the expression of respective receptor isoforms in L6 myotubes (Figure 3).

### 3.4. Effect of BSD on Glucose Uptake in L6-GLUT4myc Cells

**Figure 4:**
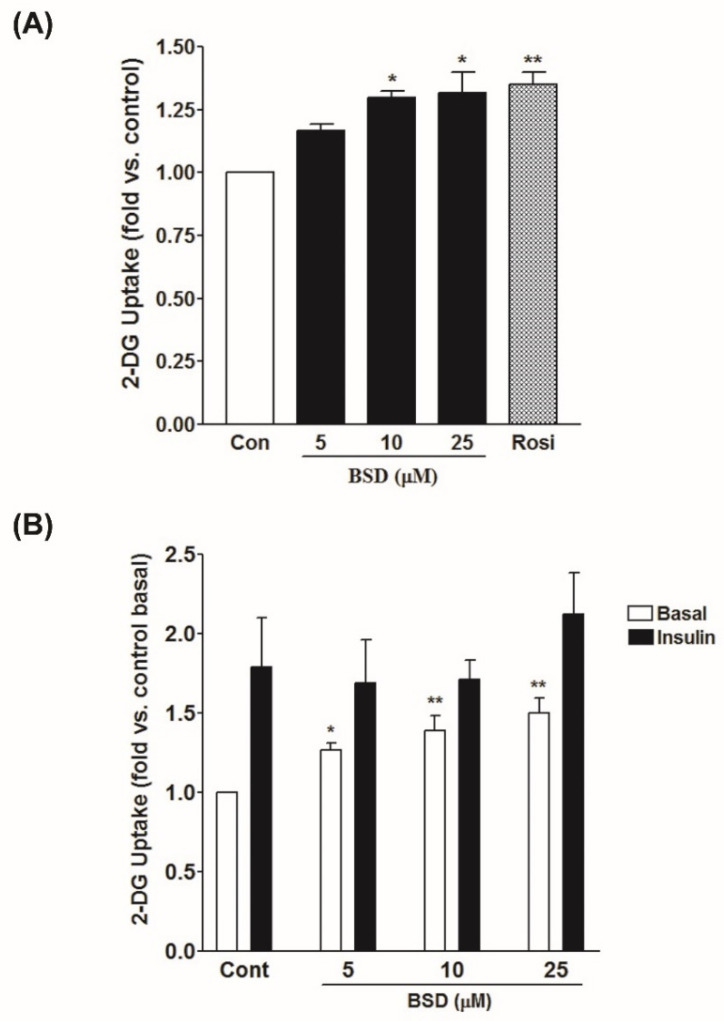
Effect of BSD on 2-deoxyglucose uptake in L6-GLUT4*myc* myotubes. Cells were incubated for 16h with increasing concentrations of BSD(**A**). After incubation, cells were left untreated (white bars) or stimulated with 100 nM insulin for 20 min (black bars) after 3h serum starvation (**B**). Glucose uptake was measured as described in the Materials and Methods section. Results are expressed as fold stimulation over control. Results shown are mean ± SE of three independent experiments. * *p* < 0.05, ** *p* < 0.01 relative to respective control condition.

To assess the effect of BSD on glucose utilization, L6-GLUT4*myc* myotubes were treated with increasing concentrations of BSD and rate of glucose uptake was monitored. Treatment with BSD potentiated the rate of glucose uptake in a concentration-dependent manner (Figure 4A). A significant increase in basal rate of glucose uptake was observed at the minimal concentration of 10 µM (1.3 ± 0.03-fold, *p* < 0.05). Acute treatment with insulin (100 nM for 20 min) enhanced glucose uptake by 1.8-fold in L6 myotubes. However, prior incubation with BSD for 16 h before insulin stimulation had no significant effect on insulin-stimulated glucose uptake, compared to control insulin-stimulated cells (Figure 4B). These findings indicate the absence of a synergistic effect between two inputs and support the likelihood that a complete insulin-dependent glucose uptake was already achieved.

### 3.5. Effect of BSD on GLUT4 Translocation in L6-GLUT4myc Cells

**Figure 5:**
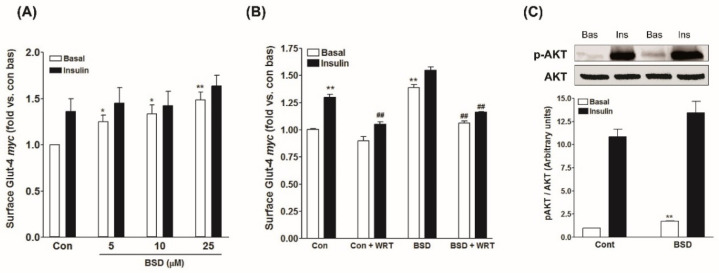
(**A**,**B**) Effect of BSD on translocation of GLUT4 to the plasma membrane in L6-GLUT4*myc* myotubes. Cells were incubated for 16 h with increasing concentrations of BSD (**A**) or with 10 µM of BSD for 16 h in the presence or absence of wortmannin (**B**). After incubation, cells were left untreated (white bars) or stimulated with 100 nM insulin for 20 min (black bars) after 3 h of serum starvation, followed by the determination of the proportion of GLUT4*myc* at the cell surface. Results are expressed as fold stimulation over control basal. Results shown are mean ± SE of three independent experiments. * *p* < 0.05, ** *p* < 0.01 relative to control basal and ^##^ *p* < 0.01 relative to respective wortmannin negative condition; (**C**) effect of BSD on phosphorylation of AKT (Ser-473) in L6-GLUT4*myc* myotubes. Cells were incubated with BSD (10 µM) for 16h, then left untreated (white bar) or stimulated with insulin (100 nM) for 10 min, followed by cell lysis and Western analysis as described in the Materials and Methods section. Shown are representative immunoblots and densitometric quantification of phospho-AKT relative to total AKT. Results of three independent experiments are presented as mean ± SE. ** *p* < 0.01 relative to control basal.

In skeletal muscle, stimulation of glucose uptake is ascribed to an enhanced rate of translocation and redistribution of the GLUT4 molecules to the cell periphery, where they facilitate the entry of the glucose to the interior of the cells for further utilization and metabolism [37]. The effect of BSD on GLUT4 translocation was monitored in L6-GLUT4*myc* myotubes. Treatment with BSD caused a substantial increase in the surface level of GLUT4*myc* molecules in a concentration-dependent manner (Figure 5a). Moreover, similar to the results of glucose uptake, prior treatment with BSD did not cause any significant effect on insulin-stimulated increase in the surface GLUT4*myc* level (Figure 5a), validating the glucose uptake data and suggesting that both insulin and BSD might activate the same signaling pathway to increase the rate of glucose utilization in skeletal muscle cells.

### 3.6. Effect of BSD on PI-3-K/Akt Mediated Signaling in Skeletal Muscle Cells

To investigate the mechanism underlying the stimulatory effect of BSD, we examined the effect on PI-3-K signaling cascade. The presence of wortmannin, a pharmacological inhibitor of PI-3-Kinase, potentially inhibited insulin-stimulated GLUT4 translocation to cell periphery in L6-GLUT4*myc* myotubes. Similarly, the effect of BSD to stimulate GLUT4 translocation was also eliminated in the presence of wortmannin (Figure 5B), suggesting the likely participation of PI-3-K-mediated signaling pathway. Furthermore, acute treatment with insulin caused a robust increase in phosphorylation of AKT at Ser-473 (Figure 5C), required for the further processing of signaling events. Likewise, treatment with BSD increased the phosphorylation of AKT at Ser-473 to a significant level under basal conditions and also synergistically enhanced the insulin-stimulated phosphorylation of AKT (Figure 5C), confirming the involvement of PI-3-K-dependent pathway in BSD-mediated biological response.

### 3.7. Effect of ER Inhibitor on BSD-Stimulated GLUT4myc Translocation

**Figure 6:**
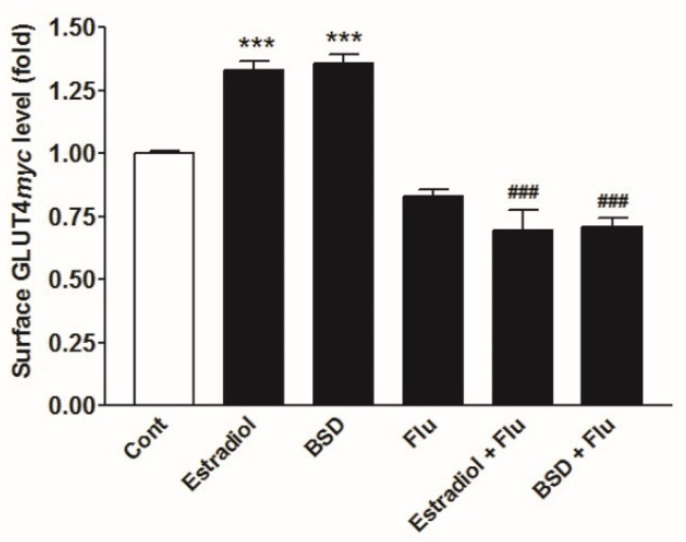
Effect of fulvestrant on BSD-induced GLUT4 translocation in GLUT4*myc* myotubes. Cells were incubated with Estradiol (10 nM) or BSD (10 µM) in the presence or absence of fulvestrant (Flu) for 16h, followed by the determination of the proportion of GLUT4*myc* at the cell surface. Results are expressed as fold stimulation over control. Results shown are mean ± SE of three independent experiments. *** *p* < 0.001 relative to control and ^###^ *p* < 0.01 relative to respective fulvestrant negative condition.

To assess the involvement of ER mediated signaling in BSD-induced GLUT4*myc* translocation, we examined the effect of BSD in the presence of ER inhibitor, fulvestrant (500 nM). The presence of fulvestrant completely reversed the Estradiol-induced translocation of GLUT4 to cell surface, to basal level (Figure 6). Likewise, BSD-induced translocation of GLUT4 was also inhibited in the presence of fulvestrant in L6-GLUT4*myc* myotubes (Figure 6). These results suggest the possible involvement of ER mediated signaling in BSD-mediated biological response to regulate glucose metabolism in skeletal muscle cells.

## 4. Discussion

Type 2 diabetes mellitus is characterized by the disturbance of glucose homeostasis, involving impaired insulin response to regulate glucose utilization in peripheral tissues, including skeletal muscle, adipose, and liver [38]. The increasing body of evidence has indicated the fundamental implication of estrogens in regulation of glycemic homeostasis [5,39]. Phytoestrogens are non-steroidal compounds of natural origin that can interact with estrogen receptors and behave like estrogen mimics. Studies in humans and experimental animals suggest that phytoestrogens, similar to estrogens, play a beneficial role in regulating nutrient metabolism and reducing the risk of obesity and diabetes. In this study, we characterized the estrogenic activity of BSD, a naturally occurring compound from the fruits of *C. sempervirens* and assessed its efficacy to regulate glucose metabolism in skeletal muscle cells.

Estrogens exert its main biological effects by binding to ERα and ERβ. However, ERα and ERβ have been shown to bind a number of structurally diverse compounds [40]. To assess the binding of BSD to ER, we used ERα-ERE luc and ERβ-ERE luc expression system in HEK-293T cells. As a validation tool for our system, Estradiol has shown to increase in reporter gene activity in both ERα-ERE luc and ERβ-ERE luc expression system. Using this assay, we have demonstrated the induction of ERE-dependent luciferase activity by BSD preferentially in the presence of ERβ. ERβ selective substances have been considered to be safe regarding unwanted proliferative side effects as they do not stimulate uterine proliferation [41].

We further investigated the specificity of BSD on some known estrogen-regulated genes in MCF-7 cells, a well-established cellular model to assess the estrogenicity of putative estrogenic compounds [42]. Estradiol has been shown to induce expression of c-*Myc* [43] and *cyclinD1* [44] in breast tissues. These genes have reported to contain non-consensus ERE sequences in their promoter region. In the current study, BSD upregulated the expression of *c-Myc* and *cyclin D1* genes in MCF-7 cells. The effects were ER dependent because the presence of fulvestrant inhibited the effect of BSD. Therefore, BSD induced estrogen-regulated genes in MCF-7 cells in a manner similar to other reported estrogen agonists. Furthermore, molecular docking studies with estrogen receptor β protein using in silico approaches suggested that BSD has significant binding affinity towards the estrogen receptors, like Estradiol. The hydrophobic interactions of the BSD molecule with the estrogen receptor protein play a crucial role in increasing the binding affinity of the molecule. Therefore, the present finding further validates that BSD can bind to the estrogen receptors.

In addition to its well established role in female physiology and reproduction, estrogens have been reported to play other physiological roles, such as the regulation of glycemic homeostasis. Skeletal muscle is the prime site for postprandial glucose utilization and is the major determinant of glucose homeostasis. In skeletal muscle, ERβ isoform expresses predominately [7], where it is reported to regulate glucose metabolism. Having established the effect of BSD to activate ERβ, we evaluated its effect on glucose utilization in skeletal muscle cells. In L6-GLUT4*myc* myotubes, treatment with BSD stimulated the rate of glucose uptake in a concentration-dependent fashion. However, prior treatment with BSD did not affect the insulin response to stimulate glucose uptake, suggesting the involvement of common signaling events in biological response of two stimuli.

In skeletal muscle, enhanced translocation and redistribution of insulin sensitive GLUT4 to cell periphery is the penultimate event for stimulation of glucose uptake inside the cell [37]. We observed that BSD exhibited concentration-dependent pattern to stimulate GLUT4 translocation in L6-GLUT4*myc* cells under basal conditions and, consistent with glucose uptake, it did not affect insulin stimulated increase in GLUT4 translocation. Results indicated that BSD has the potential to stimulate glucose uptake in skeletal muscle, likely driven by an enhanced rate of GLUT4 translocation from an internal compartment to the cell surface.

In skeletal muscle, insulin response to increase GLUT4 translocation to the cell surface is driven through phosphatidylinositol 3-kinase (PI-3-K)- and the serine/threonine-protein kinase (AKT)-mediated pathway. The PI-3-K plays a central role in metabolic action of insulin [45] and PI-3-K inhibitor like wortmannin inhibits the effect of insulin to stimulate GLUT4 translocation in skeletal muscle [29]. Similar to the insulin, the effect of BSD to stimulate GLUT4 translocation was abolished in the presence of wortmannin, suggesting the likely participation of PI-3-K-mediated signaling pathway in stimulation of GLUT4 translocation by BSD. Activation of PI-3-K leads to the progression of downstream signaling through the activation of AKT via the generation of phosphatidylinositol 3,4,5-triphosphate [46]. AKT is a serine/threonine kinase responsible for the PI-3-K-mediated metabolic responses of insulin through the phosphorylation of several substrates, including downstream protein kinases, signaling proteins, and transcription factors. Here, treatment with BSD increased AKT (Ser-473) phosphorylation in a basal state to a significant level. Altogether, findings suggest that BSD stimulates glucose utilization by augmenting the rate of GLUT4 translocation to cell periphery in skeletal muscle cells. Furthermore, the biological effects of BSD are mediated at least in part through PI-3K/AKT-dependent signaling mechanisms.

Estrogen has been shown to activate PI-3-K-mediated signaling cascade [13,47]. Given the estrogenic activity of BSD, we assessed its contribution in augmentation of PI-3-K-mediated increase in GLUT4 translocation in skeletal muscle cells. The presence of fulvestrant prevented the response of BSD to increase GLUT4 translocation in L6-GLUT4*myc*myotubes, indicating the participation of the estrogenic activity of BSD to stimulate glucose utilization in skeletal muscle cells.

## 5. Conclusions

BSD from the fruits of *C. sempervirens* exerts estrogenic activity, preferably through activating ERβ. BSD stimulates glucose uptake in L6-GLUT4*myc* myotubes by increasing the rate of translocation of GLUT4 to cell periphery. BSD exerts its effect on GLUT4 translocation through the involvement of PI-3-K/AKT-dependent pathway driven by estrogenic activity in skeletal muscle cells.

## Figures and Tables

**Table 1:** Sequence of primers (5′-3′) used for RT-PCR.

Gene	Forward Primer	Reverse Primer
H-c-Myc	TTCGGGTAGTGGAAAACCAG	CAGCAGCTCGAATTTCTTCC
H-cyclin D1	AACTACCTGGACCGCTTCCT	CCACTTGAGCTTGTTCACCA
H-18S RNA	TAGTTGGATCTTGGGAGCGG	TAGAACCGCGGTCCTATTCC
R-ERα	CAGCAGCGAGAAGGGAAACA	GGGCGGGGCTATTCTTCTTA
R-ERβ	GGACCCCAATGAACCAACG	CCTTCCTCTTCCCTATGCCC
R-18S RNA	AAACGGCTACCACATCCAAG	CCCTCTTAATCATGGCCTCA

**Table 2:** Summary of GlideScore of Estradiol and BSD.

Sr. No.	Name of Compound	Glide Score-XP (Kcal/mol)
1.	Estradiol	−8.95
2.	BSD	−9.42

## Data Availability

Data supporting the reported results will be available with the corresponding author (AK Tamrakar).

## Sample Availability

Sample of the compound BSD is available from Dr Rakesh Maurya, Medicinal and Process Chemistry Division, CSIR-Central Drug Research Institute, Lucknow, India.
